# Medical-Grade Channel Access and Admission Control in 802.11e EDCA for Healthcare Applications

**DOI:** 10.1371/journal.pone.0160052

**Published:** 2016-08-04

**Authors:** Sunghwa Son, Kyung-Joon Park, Eun-Chan Park

**Affiliations:** 1 Department of Information and Communication Engineering, DGIST, Daegu, Korea; 2 Department of Information and Communication Engineering, Dongguk University, Seoul, Korea; West Virginia University, UNITED STATES

## Abstract

In this paper, we deal with the problem of assuring medical-grade quality of service (QoS) for real-time medical applications in wireless healthcare systems based on IEEE 802.11e. Firstly, we show that the differentiated channel access of IEEE 802.11e cannot effectively assure medical-grade QoS because of priority inversion. To resolve this problem, we propose an efficient channel access algorithm. The proposed algorithm adjusts arbitrary inter-frame space (AIFS) in the IEEE 802.11e protocol depending on the QoS measurement of medical traffic, to provide *differentiated near-absolute priority* for medical traffic. In addition, based on rigorous capacity analysis, we propose an admission control scheme that can avoid performance degradation due to network overload. Via extensive simulations, we show that the proposed mechanism strictly assures the medical-grade QoS and improves the throughput of low-priority traffic by more than several times compared to the conventional IEEE 802.11e.

## Introduction

Wireless communication technologies have given rise to a paradigm shift in the communication area. By removing cables, the cost of network deployment has decreased and the portability of electronic devices has increased. Because of these benefits, wireless communications are widely adopted in diverse applications. Medical and healthcare systems are also actively focusing on convergence research with wireless communications and several companies are developing proprietary wireless solutions for healthcare applications [1–3]. Currently, there is no dedicated standard protocol for wireless healthcare applications. On the other hand, wireless local area networks (WLANs) based on IEEE 802.11 are widely deployed and most up-to-date electronic devices are equipped with a WLAN interface because of the easy installation and low cost. Therefore, we consider in this paper that WLANs are deployed in medical environments to deliver traffic for medical applications. In WLANs, however, many users and devices share a common radio channel, which has a limited capacity and is vulnerable to transmission error and interference. Therefore, it is challenging to assure a strict level of reliability and quality of service (QoS) for medical applications, referred to as *medical-grade QoS*, when adopting WLANs in healthcare systems.

In the area of WLANs, the IEEE 802.11e standard was introduced to improve the QoS for delay-sensitive applications, such as voice over Internet protocol (VoIP) and multimedia streaming. More specifically, an enhanced distributed channel access (EDCA) mechanism of the IEEE 802.11e media access control (MAC) protocol defines four access categories (ACs), each of which represents a relative level of service priority, and provides differentiated channel access depending on the ACs, i.e., a frame belonging to a high-priority AC is preferentially transmitted compared to a frame belonging to a low-priority AC.

However, this conventional EDCA only provides relative service differentiation, but it neither quantitatively assures the QoS requirements in terms of delay, loss, or throughput, nor provides their absolute guarantee. Moreover, the EDCA is prone to *priority inversion*; the transmission of a high-priority frame can be delayed owing to that of a low-priority frame, which should be avoided to assure medical-grade QoS.

In this paper, we consider the problem of assuring medical-grade QoS when the following three representative medical applications coexist: medical alarm, real-time monitoring of electrocardiogram (ECG), and non real-time medical data. Firstly, we propose an enhanced channel access scheme. The proposed scheme controls the channel access delay of low-priority traffic by tuning the value of arbitrary inter-frame space (AIFS) in IEEE 802.11e. It provides *near-absolute priority* for high-priority traffic in order to assure its medical-grade QoS, whilst at the same time, it does not unnecessarily restrict the channel access opportunity of low-priority traffic. Hence, the proposed algorithm is effective not only in preventing the priority inversion, but also in improving the overall network performance. Secondly, we propose a simple admission control scheme based on an analysis of channel capacity. The maximum allowable number of concurrent connections is determined by the capacity analysis. The proposed admission control can maintain acceptable QoS for ongoing connections by preventing network overload. Moreover, it contributes to decreasing collisions of ECG traffic owing to *delayed admission*. The main contributions can be summarized as follows:

- We propose an algorithm, called *adaptive AIFS*, which controls the channel access probability of low-priority traffic in order to guarantee the required medical-grade QoS and to improve the overall network performance.
- We derive a simple yet efficient model for the capacity of real-time ECG traffic, with which the admissible number of ECG connections is easily estimated and the admission control scheme prevents the network from being overloaded.
- We quantitatively evaluate the performance of medical-grade QoS in various aspects, by introducing a realistic measure for ECG traffic, as well as by using conventional performance measures such as delay, throughput, and collision probability.

The remainder of this paper is organized as follows: Section 2 sets out the motivation for this study and Section 3 summarizes related work. We describe the details of the proposed scheme in Section 4, and we provide extensive simulation results that confirm the effectiveness of the proposed scheme in Section 5. Our conclusion follows in Section 6.

## Motivation

### Background of IEEE 802.11e

The EDCA introduced in IEEE 802.11e was devised to differentiate the channel access opportunity per AC by setting different values of parameters associated with the channel access, for example, AIFS, contention window (CW), and transmission opportunity (TXOP), for each AC. Similar to the distributed coordination function (DCF), the basic channel access mechanism of the conventional IEEE 802.11, EDCA is basically a contention-based channel access mechanism. Under EDCA, a node that attempts to transmit a frame has to sense the channel before transmission during the period of AIFS. The length of AIFS of AC *i* is determined by the value of AIFSN as:

$${\text{AIFS}}_{i}=T_{\text{SIFS}}+{\text{AIFSN}}_{i}\cdot T_{\text{slot}},$$ (1)

where *T*_SIFS_ is the time duration of the short inter-frame space (SIFS), which is the amount of time required for a wireless interface to process a received frame and to respond with a response frame, and *T*_slot_ is a basic unit of time slot. If the channel is sensed as idle during the period of AIFS, the node performs an additional channel access procedure of backoff in order to avoid collision. The node determines an initial backoff counter value randomly between 0 and *CW*_*i*_ − 1 for each frame of AC *i*. For each idle time slot, the node decrements its backoff counter by one and it is allowed to transmit the frame if the backoff counter reaches zero. If the channel is sensed as busy during the backoff procedure, the node freezes the backoff counter and waits until the channel becomes idle. Once the channel is sensed as being idle again, the node waits for another AIFS time and resumes decreasing the frozen backoff counter. According to the *binary exponential backoff* (BEB) mechanism, the size of CW is doubled in response to the transmission failure, which can be recognized by an acknowledgement (ACK) frame from the receiver. It increases up to the maximum value of *CW*_MAX_ on consecutive transmission failures and returns to the minimum value of *CW*_MIN_ on a successful transmission. The increase of CW contributes to a decrease of collision probability, but it also increases the channel access delay, leading to the decrease of achievable throughput. As a result, by setting a smaller value of AIFSN, *CW*_MIN_, or *CW*_MAX_ to a higher-priority AC, the EDCA can give more chances of channel access for a higher-priority AC, compared with a low-priority AC. Table 1 lists default values of AIFSN, *CW*_MIN_, and *CW*_MAX_ for four ACs standardized in IEEE 802.11e. As well as AIFS and CW, TXOP is another key parameter for differentiated channel access per AC. Once a node gets the chance of channel access, it is allowed to transmit several frames backlogged in its transmission queue within the time specified by TXOP.

**Table 1:** Default values for per-AC channel access parameters in IEEE 802.11e EDCA.

Access Category	AIFSN	*CW*_MIN_	*CW*_MAX_
AC_VO (high)	2	8	16
AC_VI	2	16	32
AC_BE	3	32	1024
AC_BK (low)	7	32	1024

**Fig 1:**
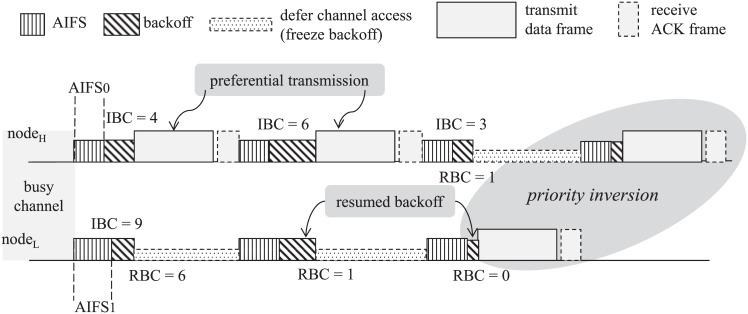
Example of priority inversion in IEEE 802.11e EDCA.

However, the prioritized channel access of EDCA cannot perfectly guarantee that a high-priority frame always gets the chance for channel access earlier than a low-priority frame, that is, the priority inversion may happen. Fig 1 illustrates a simple example of priority inversion in IEEE 802.11e EDCA. Note that the AC is determined on a per-frame basis, not on a per-node basis, i.e., a node may have different ACs for its frames. However, the example in Fig 1 considers that each node has only one AC for the sake of simplicity. Consider that the channel is shared between two STAs, one high-priority node (denoted as node_H_) whose AIFSN and *CW*_MIN_ are set to 2 and 8, respectively, and one low-priority node (denoted as node_L_) whose AIFSN and *CW*_MIN_ are set to 3 and 16, respectively, and they always have data frames to transmit. It also assume that the initial backoff counters (IBCs) of node_H_ are set to 4, 6, and 3 for its three frames, respectively, while that of node_L_ is set to 9 for its first frame. As shown in Fig 1, node_L_ defers the transmission of its first low-priority frame until node_H_ finishes transmitting two high-priority frames. It is worthwhile to note that, during the transmission time of two high-priority frames, the residual backoff counter (RBC) of node_L_ decreases from the initial value of 9 to 6, and from the frozen value of 6 to 1, respectively, according to Eq (1). Consequently, the RBC of node_L_ becomes smaller than the IBC of node_H_, and the first frame of node_L_ can be transmitted sooner than the third frame of node_H_. This example confirms that even larger values of AIFSN and CW in the low-priority AC eventually lead to a priority inversion, which originates from the property of freezing/releasing the backoff counter and is unavoidable in the contention-based channel access. We can expect that the probability of priority inversion increases as the number of low-priority nodes increases. Furthermore, we can consider a possible solution to alleviate the priority inversion: making a large difference between the values of AIFSN or CW for different ACs. However, this naive approach unnecessarily increases the channel access delay of low-priority frames and decreases the throughput accordingly.

### Problem statement via preliminary simulation

We perform a preliminary simulation to confirm that a straightforward application of IEEE 802.11e to medical traffic is neither effective nor sufficient to assure medical-grade QoS.

We consider three coexisting medical applications, medical alarm, real-time ECG monitoring, and non real-time (NRT) medical data. As shown in Table 2, we map these applications to AC_VO, AC_VI, and AC_BE, respectively, based on their priorities and the convention of IEEE 802.11e standard. We consider the transport-layer protocol of medical alarm and real-time ECG monitoring to be user datagram protocol (UDP) for fast transmission, while that of NRT data is transmission control protocol (TCP) for reliable transmission. In addition, we assume that each node has one class of medical application. The network consists of five nodes with medical alarm, 20 nodes with NRT data, and a variable number of nodes with real-time ECG monitoring ranging from 1 to 25. The simulation parameters are set to the default values of IEEE 802.11e EDCA, as given in Table 1. Details of the simulation configuration can be found in Section 5.

**Table 2:** Mapping medical applications to IEEE 802.11e ACs.

Access Category	Priority	Medical application
Voice (AC_VO)	Highest	Medical alarm
Video (AC_VI)	High	Real-time monitoring
Best Effort (AC_BE)	Medium	non-real-time medical data
Background (AC_BK)	Lowest	Non-medical applications

**Fig 2:**
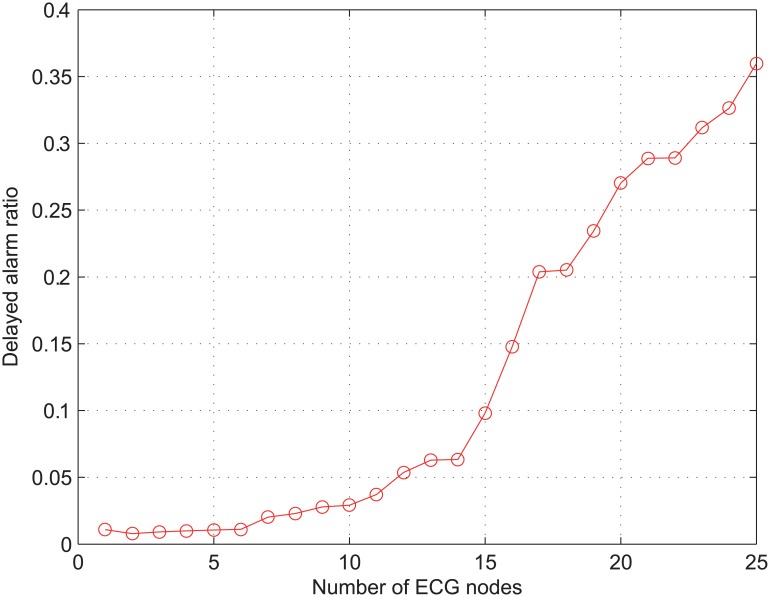
Ratio of delayed alarm packets over total received alarm packets.

Fig 2 illustrates the ratio of delayed alarm packets (denoted as *γ*_*alarm*_) with respect to the number of real-time ECG monitoring nodes (denoted as *N*_*ECG*_). Here, we consider that an alarm packet is delayed if its delay is longer than the typical maximum allowable value of 200 msec [4]. We can easily observe from Fig 2 that *γ*_*alarm*_ significantly increases as *N*_*ECG*_ increases; for example, *γ*_*alarm*_ exceeds 0.1 as long as *N*_*ECG*_ > 15 and it is about 0.36 when *N*_*ECG*_ = 25. This result confirms that the conventional IEEE 802.11e EDCA is not capable of fully protecting the high-priority traffic from the low-priority traffic.

**Fig 3:**
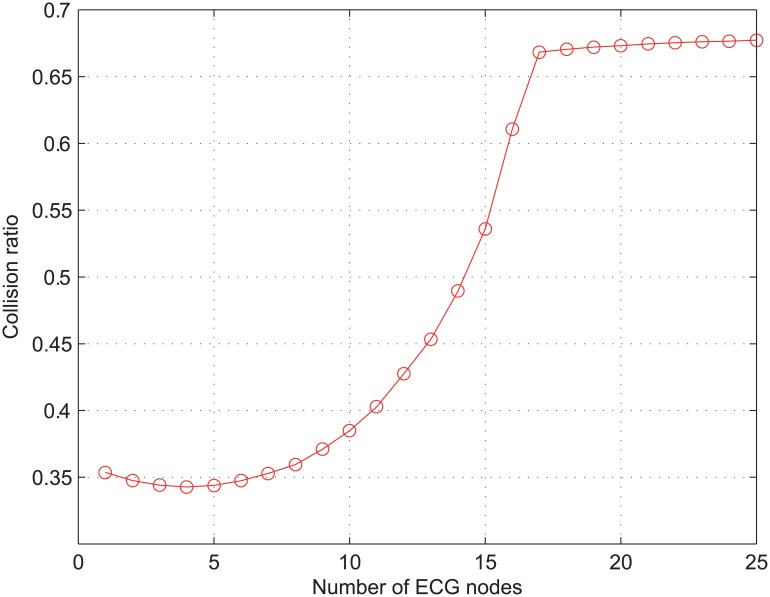
Collision ratio of the overall network traffic.

The poor performance of *γ*_*alarm*_ in Fig 2 can be explained from two points: (*i*) priority inversion and (*ii*) collision. As shown in Table 1, medical alarm assigned as AC_VO and real-time monitoring traffic as AC_VI have the same value of AIFSN. Therefore, the service of these two types of traffic is differentiated only in terms of CW, and the level of service differentiation between them is not high enough to avoid the priority inversion as discussed in Section 2.1. In addition, the probability of priority inversion increases with respect to the increase of *N*_*ECG*_. The collision is another reason for the performance degradation of *γ*_*alarm*_. Fig 3 shows the collision ratio of the overall network traffic, which is denoted as *γ*_*coll*_ and defined as the total number of collided packets divided by the total number of transmitted packets. As long as *N*_*ECG*_ < 8, *γ*_*coll*_ does not remarkably change from the value of 0.35. This is because the network is not overloaded with a small number of nodes transmitting the real-time ECG traffic and 20 nodes with NRT data traffic adjust their transmission rates according to a TCP congestion control mechanism. However, when *N*_*ECG*_ increases from 10 to 17, *γ*_*coll*_ sharply increases from 0.38 to 0.67. The increase of *γ*_*coll*_ in this range results from the small value of *CW*_MIN_. Once *N*_*ECG*_ exceeds 17, *γ*_*coll*_ is almost immune to the increase of *N*_*ECG*_. This result implies that the frequent collisions increase the value of CW close to its maximum so that *γ*_*coll*_ is hardly affected by the increase of *N*_*ECG*_.

In summary, the results in Figs 2 and 3 validate the limited performance of the conventional IEEE 802.11e in dealing with the medical traffic.

## Related work

Recently, the convergence research of healthcare and wireless communication is being actively performed [5–7]. As a part, there are numerous studies on improving the performance of the conventional IEEE 802.11e standard, especially from the QoS perspective [8–17]. The previous studies mostly control CW, AIFS, or TXOP to improve the QoS of real-time traffic. Essentially, the prioritized channel access in the IEEE 802.11e MAC protocol can be achieved at the cost of performance degradation of low-priority traffic [9] because the IEEE 802.11e protocol employs pre-defined parameters without considering the network condition. The following paragraphs introduce various existing approaches for controlling CW, TXOP, and AIFS, to improve performance of IEEE 802.11e.

Romdhani et al. [10] proposed the Adaptive Enhanced Distributed Coordination Function (AEDCF), which adjusts the value of CW based on application requirements and estimated collision rate. Deb and Hartman [11] provided an analytical model of the network state as a function of MAC parameters and proposed a distributed algorithm for optimizing the value of CW in order to improve QoS and maximize the channel utilization. The algorithm proposed by S. Pudasaini and S. Shin [12] aims to avoid QoS degradation resulting from the priority inversion. In this algorithm, the backoff counter of low-priority traffic is stochastically determined such that it becomes larger than the maximum CW size of high-priority traffic. Although this approach can minimize the priority inversion, it may increase collisions among nodes that have the same priority traffic. It is worthwhile to note that, as indicated in Fig 1, the backoff procedure is frozen/resumed on the channel occupation/release by the other nodes. On the contrary, a node should defer the channel access during the AIFS time before every backoff procedure. Therefore, the approach of controlling CW is less effective at preventing the priority inversion for assuring medical-grade QoS, compared to the approach of controlling AIFS.

Freitag et al. [13] proposed an algorithm for tuning CW and TXOP, in order to solve the problem of delay asymmetry between uplink and downlink connections while maintaining QoS. Arora et al. [14] proposed the idea of adaptive TXOP allocation to increase channel efficiency and assure temporal fairness among stations. This scheme adapts the length of TXOP according to traffic requirements and channel conditions within the predefined TXOP interval. Andreadis and Zambon [15] introduced a Dynamic TXOP (DTXOP) algorithm to improve QoS by providing fair resource allocation in both uplink and downlink directions. They also proposed an admission control integrated with DTXOP for QoS support under heavy traffic load. Ghazvini et al. [16] derived an analytical model of IEEE 802.11e EDCA and proposed a game theoretic approach for determining TXOP dynamically in order to improve QoS and network performance. These approaches based on TXOP control are inherently effective to improve channel efficiency and fairness by decreasing channel access overheads and enforcing comparable channel occupation time. However, they are not suitable for our medical scenario because TXOP mainly deals with burst data traffic whose characteristics are quite different from those of medical traffic.

The recent study in [17] aims to provide medical-grade QoS by providing high-priority traffic with an absolute priority of channel access. Its main idea is to extend the value of low-priority AIFS so that it becomes larger than the maximum defer time of high-priority traffic, i.e.:

$${\text{AIFS}}_{L}={\text{AIFS}}_{H}+CW_{\text{MAX},H}\cdot T_{\text{slot}},$$

where AIFS_*L*_ and AIFS_*H*_ denote the AIFS of low- and high-priority traffic, respectively, and *CW*_MAX,*H*_ is the maximum CW size of high-priority traffic. Therefore, high-priority traffic always has a preferred opportunity of channel access; that is, it can completely prevent the priority inversion. However, this approach may unnecessarily degrade the performance of low-priority traffic because the low-priority node should defer its channel access for a considerable time even if there is no high-priority traffic. Apart from QoS assurance, the energy efficiency is one of the primary issues in mobile devices and wireless communication systems. Therefore, a suitable energy-efficient resource management should be considered [18, 19].

Our study is different from the previous studies and it has several advantages and desirable properties in the following aspects:

- We consider a realistic traffic model for healthcare application (e.g., ECG monitoring) and introduce a novel measure to quantitatively evaluate its performance.
- We adopt the approach of AIFS control along with a simple admission control to assure medical-grade QoS, since the approach of CW control is not effective to avoid the priority inversion and the approach of TXOP control is not suitable to deal with real-time medical traffic.
- As well as providing medical-grade QoS, our mechanism avoids unnecessary performance degradation of low-priority traffic.

## Proposed algorithm

In this section, we propose a medical-grade channel access scheme that can achieve the following two goals: (*i*) assuring *differentiated near-absolute priority* for medical traffic and (*ii*) improving efficiency of channel sharing. It should be noted that there is a certain level of tradeoff between these two goals. If we intend to absolutely protect high-priority traffic from low-priority traffic to avoid priority inversion, the channel utilization will be inevitably degraded. Therefore, the proposed scheme is designed to provide medical traffic with *near-absolute priority* so that the medical-grade QoS can be maintained at an acceptable level, whilst at the same time, the channel access of low-priority traffic is not unnecessarily restricted to improve the channel utilization. Moreover, the proposed scheme is intended to support the near-absolute priority for medical traffic in a *differentiated* way depending on the urgency and importance of the medical traffic. To achieve these goals, the proposed mechanism integrates two schemes, an adaptive AIFS scheme and admission control, each of which is described in the following subsections.

### Adaptive AIFS scheme

#### Design rationale and measurement of medical-grade QoS

**Fig 4:**
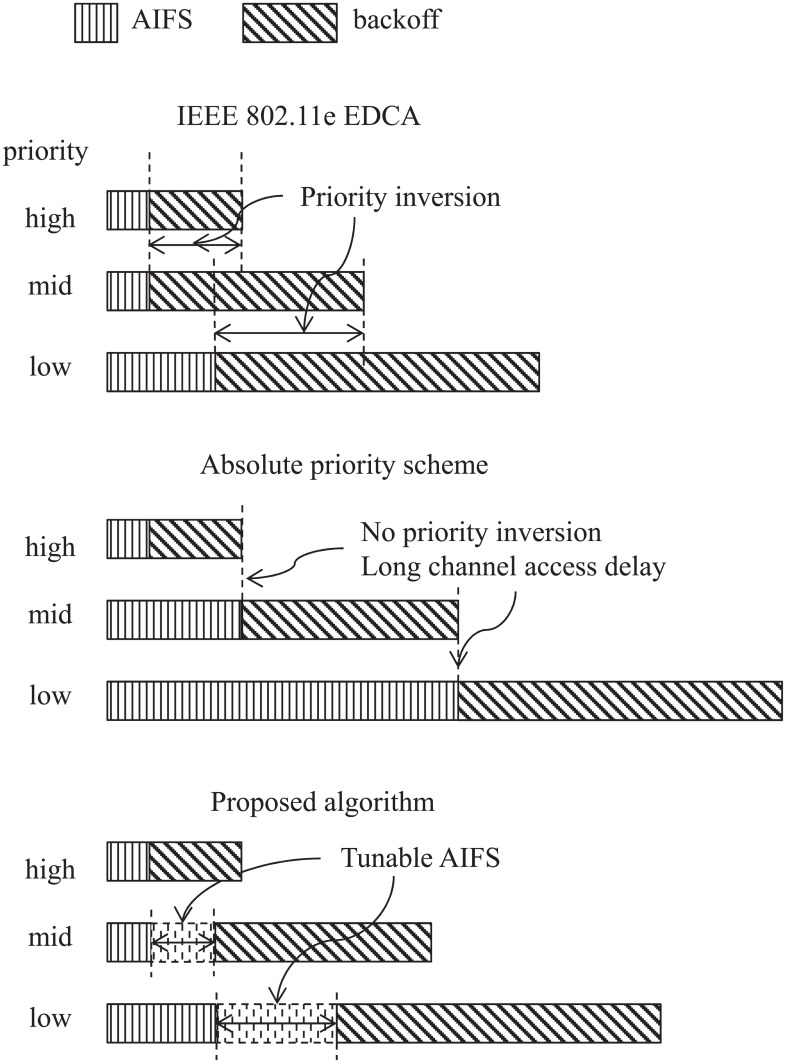
Conceptual comparison of IEEE 802.11e EDCA, absolute priority scheme, and the proposed adaptive AIFS scheme in terms of AIFS and CW.

The basic concept of the adaptive AIFS scheme can be explained from Fig 4, which compares it with IEEE 802.11e EDCA and absolute priority scheme [17]. As illustrated in Fig 4, the IEEE 802.11e EDCA is vulnerable to priority inversion, which may happen when the range of backoff counter for higher-priority traffic overlaps with that for lower-priority traffic. On the other hand, the absolute priority scheme is completely free from the priority inversion at the cost of a long channel access delay for lower-priority traffic. The proposed scheme overcomes the drawbacks of these two existing schemes by adaptively controlling the value of AIFS based on the measurement of medical-grade QoS. The key idea of AIFS adaptation is quite simple, as follows:

1. Increase the value of AIFS of low-priority traffic if the QoS requirement of high-priority traffic is liable to be violated.
2. Keep the current value of AIFS as long as the medical-grade QoS is maintained at an acceptable level.
3. Decrease the value of AIFS of low-priority traffic if the level of medical-grade QoS is high enough to tolerate a slight degradation.

The underlying rationale of controlling AIFS, instead of CW, is that AIFS is more effective at differentiating the channel access than CW, as discussed in Section 3.

Now, we describe how to measure the medical-grade QoS, which provides a basis to adapt the value of low-priority AIFS. In order to establish a proper QoS measurement for medical application, we need to consider its usage, characteristics, and requirements as follows:

- **Medical alarm**: Typical examples of this application are a telemetry alarm in emergency rooms and an infusion pump warning in operating or patient rooms. The traffic for these applications are generated in an unscheduled manner. This is the most critical application and should be transmitted with the highest priority. Any packet loss should be completely avoided but it can tolerate a delay up to a certain level (e.g., less than 100 msec). Therefore, the delay can be a proper QoS metric for this application.
- **Real-time ECG monitoring**: This application continuously measures and transfers the timing and duration of the electrical phase of a patient’s heartbeats. It can be used to diagnose/discover abnormal heart symptoms. This application generates packets periodically and tolerates a small delay. Considering the property of real-time traffic, we establish its QoS metric as the fraction of packets whose delay exceeds the tolerable value among the total packets received. Furthermore, we consider that the fraction of delayed packets should be maintained lower than a given tolerable value (e.g., less than 1%), to avoid misdiagnosis.
- **NRT data delivery**: Examples of this application include an electronic medical record (EMR) or bar code medication administration (BCMA). Unlike the other applications, this is not a delay-sensitive application, but it requires reliable transmission; thus, it is delivered by TCP and has the lower priority than the others.

Next, we devise a practical mechanism to measure the delay, because the medical-grade QoS is mostly related to the delivery delay, as discussed above. We consider an infrastructure mode WLAN where all the WLAN nodes are associated with an access point (AP) and they transmit and receive frames to and from the AP, respectively. In addition, we assume that the time is synchronized between the AP and all the nodes, which can be achieved by the timing synchronization function specified in the IEEE 802.11 standard. The AP periodically broadcasts a beacon frame that includes a time stamp. The value of the time stamp is determined by the local time of the AP and its unit is one microsecond. On receiving the beacon frame, the nodes synchronize their local timers with the value of the time stamp in the beacon frame. We consider that the WLAN-equipped medical devices transmit packets to the AP. Whenever a packet is generated to transmit in the medical device, the device maintains the packet generation time and attaches this timing information to the MAC header. When receiving the packet from the medical device, the AP calculates the delivery delay by extracting the packet generation time from the MAC header. Note that the delivery delay includes queuing delay, channel access delay, and retransmission delay. For the purpose of measuring delivery delay, a field containing timing information should be added in the MAC header. We consider that the size of this field does not exceed two bytes in typical configurations, which is smaller than the size of packet by at least two orders of magnitude. In this way, the AP can measure the delivery delay of medical traffic without significant overhead.

The AP adjusts the value of AIFS based on the measured QoS metric. The details of the adaptive AIFS scheme are described in Algorithm 1 and the notations of QoS metrics, control variables, and design parameters are summarized in Table 3. The proposed scheme deals with two types of medical applications (alarm and ECG monitoring) in a differentiated way because of their different traffic characteristics and QoS requirements.

**Table 3:** Notations of QoS metrics, control variables, parameters used in the adaptive AIFS scheme.

Category	Notation	Description	Value
QoS metric	delay_alarm	Delay of medical alarm traffic	
	cnt_QoS_violat	Number of delayed alarm packets during MNT_INTV_ALR	
	dlyd_ECG_rto	Ratio of delayed ECG packets during MNT_INTV_ECG	
Control variables	aifsn_ECG	Value of AIFSN for ECG traffic	
	aifsn_DATA	Value of AIFSN for data traffic	
System parameters	INIT_AIFSN_ECG	Initial value of AIFSN for ECG traffic	2
	INIT_AIFSN_DATA	Initial value of AIFSN for data traffic	3
	CW_MAX_ALR	Maximum CW value of alarm traffic	16
	CW_MAX_ECG	Maximum CW value of ECG traffic	32
Design parameters	MAX_DLY_ALR	Criterion of delay_alarm for critical QoS degradation	200 msec
	TLR_DLY_ALR	Tolerable delay of alarm traffic	100 msec
	MNT_INTV_ALR	Monitoring interval of alarm traffic to measure cnt_QoS_violat	1 sec
	MNT_INTV_ECG	Monitoring interval of ECG traffic to measure dlyd_ECG_rto	1 sec
	MAX_DLY_ECG	Maximum allowable delay of ECG packet	200 msec
	MAX_ECG_RTO	Maximum threshold value of dlyd_ECG_rto	0.01
	MIN_ECG_RTO	Minimum threshold value of dlyd_ECG_rto	0.005

**Algorithm 1** Pseudocode of the proposed adaptive AIFS algorithm.

/* Initialize variables */

cnt_QoS_violat = 0; /* number of delayed alarm packets */

num_ECG_pkt = 0; /* number of ECG packets received */

num_delayed_ECG = 0; /* number of delayed ECG packets */

/* On receiving alarm or ECG packet*/

**if** get_pkt_type(packet) == ALARM **then**

 delay_alarm = calculate_delay(packet);

 **if** delay_alarm ≥ MAX_DLY_ALR **then**

  /* Severe QoS degradation of alarm traffic */

  aifsn_ECG = CW_MAX_ALR;

  aifsn_DATA = CW_MAX_ECG;

  cnt_QoS_violat++;

 **else if** delay_alarm ≥ TLR_DLY_ALR **then**

  /* Undesirable QoS degradation of alarm traffic */

  aifsn_ECG = min (aifsn_ECG++, CW_MAX_ALR);

  aifsn_DATA = min (aifsn_DATA++, CW_MAX_ECG);

  cnt_QoS_violat++;

 **end if**

**else if** get_pkt_type(packet) == ECG **then**

 delay_ECG = calculate_delay(packet);

 num_ECG_pkt++;

 **if** delay_ECG ≥ MAX_DLY_ECG **then**

  num_delayed_ECG++;

 **end if**

**end if**

/* On every MNT_INT_ALARM or MNT_INT_ECG */

**if** cnt_QoS_violat == 0 **then**

 /* Acceptable QoS of alarm traffic */

 aifsn_ECG = max (aifsn_ECG–, INIT_AIFSN_ECG);

 aifsn_DATA = max (aifsn_DATA–, INIT_AIFSN_DATA);

**end if**

dlyd_ECG_rto = num_delayed_ECG / num_ECG_pkt;

**if** dlyd_ECG_rto ≥ MAX_ECG_RTO **then**

 /* Degraded QoS of ECG traffic */

 aifsn_DATA = min (aifsn_DATA++, CW_MAX_ECG);

**else if** dlyd_ECG_rto < MIN_ECG_RTO **then**

 /* Acceptable QoS of ECG traffic */

 aifsn_DATA = max (aifsn_DATA–, INIT_AIFSN_DATA);

**end if**

/* Reset variables */

cnt_QoS_violat = 0;

num_ECG_pkt = 0;

num_delayed_ECG = 0;

#### Control rule of AIFS in response to the QoS measurement of medical alarm

Firstly, we focus on the control rule of AIFS depending on the delay of medical alarm. Here, we introduce two thresholds, MAX_DLY_ALR and TLR_DLY_ALR. The former is used as a criterion of severe QoS degradation of the medical alarm, while the latter denotes its tolerable delay. Taking the characteristics and requirements of medical alarm [4] into account, MAX_DLY_ALR and TLR_DLY_ALR are set to 200 msec and 100 msec, respectively. Whenever a packet of medical alarm is received, the AP measures its delay, denoted as delay_alarm. Depending on the value of delay_alarm, there are three cases for adjusting the value of AIFS:

(C1) Critical QoS degradation:
  delay_alarm ≥ MAX_DLY_ALR
(C2) Undesirable QoS degradation:
  TLR_DLY_ALR ≤ delay_alarm < MAX_DLY_ALR
(C3) QoS assurance:
delay_alarm < TLR_DLY_ALR

We focus on the first case (C1). Because this case should be avoided as soon as possible, aifsn_ECG and aifsn_DATA are immediately increased to their upper limits of CW_MAX_ALR and CW_MAX_ECG, respectively. This control rule is similar to the absolute priority scheme [17] (see Fig 4) and maximizes the channel access opportunity of alarm traffic. In order to quickly inform medical devices of these changes in aifsn_ECG and aifsn_DATA, we employ a dedicated signaling mechanism between the AP and medical devices. For this purpose, we design a new broadcasting control frame containing the values of aifsn_ECG and aifsn_DATA. On receiving this broadcasting frame from the AP, the medical devices update their local values of AIFSN to the values in the broadcasting frame. Consequently, the medical devices decrease their channel access attempts to deliver lower-priority traffic (ECG monitoring and NRT data), and hence, the delay of high-priority alarm traffic can be decreased.

We consider the second case (C2). Whenever AP detects that delay_alarm exceeds TLR_DLY_ALR, aifsn_ECG and aifsn_DATA are increased by one. Compared to the first case (C1), this case is less urgent to satisfy the medical-grade QoS of alarm. Therefore, the values of aifsn_ECG and aifsn_DATA are gradually increased up to their upper limits of CW_MAX_ALR and CW_MAX_ECG. We consider that these updated values of AIFSN can be delivered to the medical devices via the periodic beacon frames, instead of the broadcasting control frame. For this purpose, the beacon frame needs to be slightly modified. Considering that the WLAN system is dedicatedly deployed in healthcare environment, this approach is feasible. Note that the typical period of beacon frame is 100 msec, which is comparable to the value of TLR_DLY_ALR. Therefore, the delivery of AIFSN information via beacon frame in this case can effectively decrease the signaling overhead without significantly increasing the signaling delay.

In contrast to the previous two cases of (C1) and (C2), the devices with low-priority traffic in the case of (C3) do not have to decrease their channel access attempts because the medical-grade QoS of the high-priority traffic attains an acceptable level. In this case, there is the potential of improving channel utilization by increasing the channel access opportunity of low-priority traffic while maintaining the near-absolute priority for medical alarm. Here, we introduce a new measurement, cnt_QoS_violat, which is the number of alarm packets whose delay exceeds TLR_DLY_ALR during every monitoring interval (denoted as MNT_INTV_ALR). If cnt_QoS_violat is zero, aifsn_ECG and aifsn_DATA are decreased by one. The lower limits of aifsn_ECG and aifsn_DATA are set to their initial values of INIT_AIFSN_ECG and INIT_AIFSN_DATA, respectively. In this way, the channel access probability of lower-priority traffic can increase. Otherwise if cnt_QoS_violat > 0, aifsn_ECG and aifsn_DATA keep their current values, not to debase the QoS of alarm traffic. Similarly to the case of (C2), the information of aifsn_ECG and aifsn_DATA can be delivered via beacon frames for every MNT_INTV_ALR. If we set MNT_INTV_ALR to a large/small value, lower-priority traffic (ECG monitoring and NRT data) can be delivered in a more conservative/aggressive way. Since the medical alarm has the highest priority and its traffic is generated unexpectedly, we consider that MNT_INTV_ALR needs to be in the order of ten beacon intervals.

#### Control rule of AIFS in response to the QoS measurement of ECG monitoring

Next, we describe the control rule of AIFS depending on the QoS measurement of ECG monitoring traffic. As described in Section 4.1.1, we introduce the QoS metric of ECG traffic, dlyd_ECG_rto, defined as the number of ECG packets whose delay exceeds the maximum tolerable value of MAX_DLY_ECG divided by the number of total ECG packets received during the interval of MNT_INTV_ECG. We consider that the typical packet generation interval of real-time ECG traffic is in the order of a hundred milliseconds. We also consider that the received ECG packet whose delay is larger than its generation interval is useless, because of the nature of real-time traffic. Under these rationales, we set MAX_DLY_ECG and MNT_INTV_ECG to 200 msec and 1 sec, respectively. It is important to note that the QoS metric of alarm traffic is the delay of an individual packet, whilst that of ECG traffic is the ratio of delayed packets among several packets. This is because the alarm traffic has the highest priority and it cannot tolerate any packet loss, while the real-time ECG traffic can tolerate a small probability of packet loss (e.g., less than 1%).

Depending on the measurement of dlyd_ECG_rto, the AIFS of lower-priority traffic, aifsn_DATA, is adjusted. In a similar way to the alarm traffic, we introduce two thresholds for delayed ECG ratio, MAX_ECG_RTO and MIN_ECG_RTO. We consider that it is desirable that dlyd_ECG_rto is maintained between these two thresholds, from the viewpoints of attaining near-absolute priority for ECG traffic and improving channel utilization. Firstly, if dlyd_ECG_rto ≥ MAX_ECG_RTO, aifsn_DATA is increased by one up to its maximum value of CW_MAX_ECG so that the channel access opportunity of lower-priority data traffic decreases and dlyd_ECG_rto can decrease below MAX_ECG_RTO. Secondly, if dlyd_ECG_rto lies between MAX_ECG_RTO and MIN_ECG_RTO, the medical-grade QoS of ECG traffic is acceptable and the value of aifsn_DATA is not changed. Lastly, if dlyd_ECG_rto is smaller than MIN_ECG_RTO, aifsn_DATA is decreased by one up to its initial value of INIT_AIFSN_DATA, and thus, the channel utilization can be improved. For every interval of MNT_INTV_ECG, the value of aifsn_DATA is updated and delivered to the devices via beacon frames.

### Admission control with network capacity analysis

The objective of the adaptive AIFS algorithm in Section 4.1 is to guarantee the near-absolute priority for medical-grade QoS while enhancing the network throughput. However, as plotted in Fig 3, it is obvious that the network cannot support the medical-grade QoS once the network is overloaded. Network overload can lead to overall performance degradation of medical applications, which should be strictly avoided in medical situations. To prevent this critical incident, we introduce an admission control based on the capacity analysis of the medical application. Among the three medical applications considered in this study, admission control is applied only to the real-time ECG monitoring application because of the following reasons: The highest-priority medical alarm should be delivered timely, regardless of network load, so it is not desirable to deny the service of medical alarm even when the network is highly loaded. On the other hand, the low-priority NRT data is served in a best-effort manner by using TCP and its transmission rate is autonomously adjusted by the TCP congestion control mechanism. Therefore, it is useless to apply the admission control to the NRT data application.

#### Capacity analysis of ECG application

The essence of admission control is to analyze the network capacity of real-time ECG applications. The analysis in this work follows a similar line for the saturated condition as in [20]. The probability of each network state *π*_*i*_ is defined as the fraction of time that node *i* is transmitting where *n* nodes are in the network of the same carrier sensing range. The fraction of idle channel time is represented as *π*_0_. In addition, let *θ*_*i*_ = *E*[*T*_*i*_]/*E*[*B*_*i*_] denote the ratio of the expected transmission time *E*[*T*_*i*_] to the expected backoff time *E*[*B*_*i*_] for transmitting node *i*.

We define the number of transmissions by node *i* as *c*_*i*_(*t*). For *c*_*i*_(*t*) instances of transmission, node *i* performs the backoff procedure *c*_*i*_(*t*) times. When the channel is idle, all the nodes that have packets to transmit are permitted to decrease their backoff counters. Therefore, the ratio *π*_*i*_/*π*_0_ can be expressed as:

$$\begin{array}{cc} \frac{\pi_{i}}{\pi_{0}} & =\lim_{t\to \infty }\frac{\frac{1}{t}\sum_{j=1}^{c_{i}\left(t\right)}T_{i}\left(j\right)}{\frac{1}{t}\sum_{j=1}^{c_{i}\left(t\right)}B_{i}\left(j\right)}=\lim_{t\to \infty }\frac{\frac{1}{c_{i}\left(t\right)}\sum_{j=1}^{c_{i}\left(t\right)}T_{i}\left(j\right)}{\frac{1}{c_{i}\left(t\right)}\sum_{j=1}^{c_{i}\left(t\right)}B_{i}\left(j\right)}\\ & =\frac{E[T_{i}]}{E[B_{i}]}=\theta_{i}, \end{array}$$ (2)

where *B*_*i*_(*j*) is the length of the *j*-th backoff interval of node *i* and *T*_*i*_(*j*) is the length of the *j*-th transmission of node *i*. From Eq (2), a system of linear equations is given as follows:

$$\pi_{0}=\frac{\pi_{1}}{\theta_{1}}=\frac{\pi_{2}}{\theta_{2}}=\ldots =\frac{\pi_{n}}{\theta_{n}}.$$ (3)

In our scenario, we consider that the ECG monitoring application generates its packet periodically, that is, the node with the ECG application does not always have packets to transmit. In order to cope with this property of the ECG application, we now employ the unsaturated version of the analysis in [20]. In an unsaturated node, it decreases its backoff counter only when it has a packet to transmit. Let us define *ρ*_*i*_ (0 < *ρ*_*i*_ ≤ 1) as the probability that the node *i* performs the backoff procedure under the condition that the channel is idle. Then, the effective backoff time in the unsaturated node *i* is reduced by *ρ*_*i*_, compared to the saturated case, and *θ*_*i*_ needs to be changed from *π*_*i*_/*π*_0_ in the saturated case to *π_i_*/(*ρ_i_π*_0_) in the unsaturated case. Introducing a new variable *γ*_*i*_ = *ρ*_*i*_*θ*_*i*_, the system of Eq (3) is changed to:

$$\pi_{0}=\frac{\pi_{1}}{\gamma_{1}}=\frac{\pi_{2}}{\gamma_{2}}=...=\frac{\pi_{n}}{\gamma_{n}}.$$ (4)

From the condition of $\sum_{i=0}^{n}\pi_{i}=1$, Eq (4) can be solved as follows:

$$\begin{array}{cc} \pi_{0} & =\frac{1}{1+\sum_{i=1}^{n}\gamma_{i}},\\ \pi_{i} & =\frac{\gamma_{i}}{1+\sum_{i=1}^{n}\gamma_{i}}. \end{array}$$ (5)

We consider that a MAC protocol data unit of node *i* consisting of a MAC header (including frame check sequence) and a payload is transmitted at the rate of *r*_*i*_, while the physical(PHY)-layer header (including preamble) and ACK frame are transmitted at the rate of *r*_*min*_(≤ *r*_*i*_), regardless of *r*_*i*_, for the reliable transmission. Let us denote *T*_*PH*_, *T*_*MH*_, *T*_*PL*_, and *T*_*ACK*_ as the time required to transmit the PHY header, MAC header, payload, and ACK frame, respectively. Then, the effective transmission rate of node *i* to transmit a packet, denoted as $\bar{r_{i}}$, can be represented as:

$${\bar{r}}_{i}=w_{i}r_{i}+\left(1-w_{i}\right)r_{min},$$

where *w*_i_ = (*T_MH_* + *T_PL_*)/(*T_PH_* + *T_MH_* + *T_PL_* + *T_ACK_*). In calculating $\bar{r_{i}}$, we do not consider *T*_SIFS_, which is negligible compared to *T*_*PL*_. Finally, the achievable throughput of unsaturated node *i*, denoted as *x*_*i*_, is given from Eq (5) as:

$$x_{i}=\pi_{i}{\bar{r}}_{i}=\left(\frac{\gamma_{i}}{1+\sum_{i=1}^{n}\gamma_{i}}\right){\bar{r}}_{i}.$$ (6)

**Fig 5:**
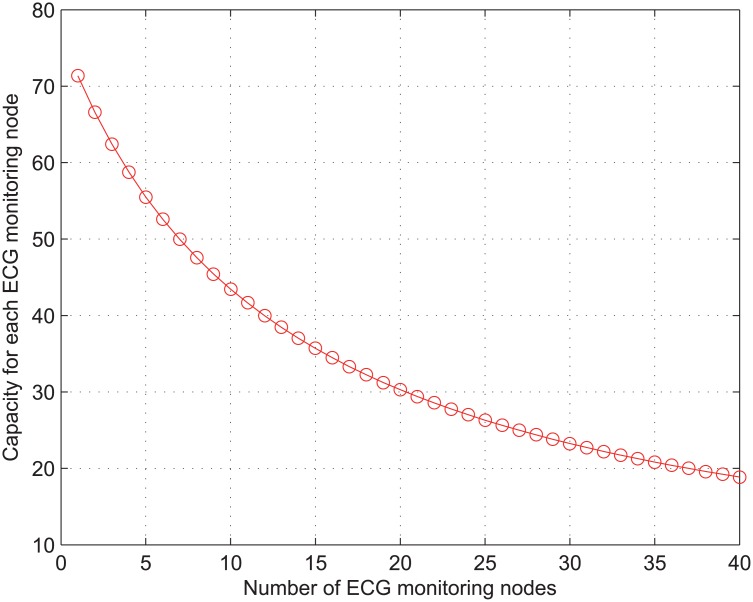
The capacity of ECG monitoring traffic.

Fig 5 shows the achievable throughput of one ECG node calculated from Eq (6) when the number of ECG nodes increases from 1 to 40. Here, both values of *r*_*i*_ and *r*_*min*_ are set to 1 Mb/s for all the ECG nodes. In our study, we consider that the ECG monitoring application generates its packet at a rate of 26 Kb/s [4]. As long as the number of ECG monitoring nodes is not larger than 25, the achievable throughput of the ECG node exceeds 26 Kb/s, i.e., it satisfies the requirement of medical-grade QoS in the ECG application. Consequently, we can conclude that the network capacity for ECG application is given as 25 connections when *r*_*i*_ is set to 1 Mb/s.

#### Design of admission control for ECG application

Based on this capacity analysis in Section 4.2.1, we design a simple admission control for ECG application. We consider that a medical device transmits an admission request message to the AP for establishing a new ECG connection, and the AP replies to this request by transmitting an admission response message to the node. Let us define *n*_*ECG*_ and $N_{ECG}^{MAX}$ as the number of on-going ECG connections and its maximum allowable number calculated based on the capacity analysis (e.g., 25). Whenever receiving the admission request message, the AP accepts this request as long as $n_{ECG}<N_{ECG}^{MAX}-\Delta N_{ECG}$ , where Δ*N*_*ECG*_ is a positive integer value. Here, Δ*N*_*ECG*_ is introduced for two reasons: (*i*) to reserve the network capacity for serving the highest-priority alarm traffic and (*ii*) to take the modeling error of capacity analysis into account. After the ECG connection is admitted, the AP updates the value of *n*_*ECG*_. If the AP does not receive any ECG packet from the established connection for a given time, which is sufficiently longer than the packet generation interval of the ECG application, then the AP assumes that the connection is terminated and decreases *n*_*ECG*_ by one. In the procedure of admission control, the AP does not perform any operations of calculating the probabilities or rates in Eqs (2)–(6) to estimate $N_{ECG}^{MAX}$, which can be determined based on the traffic model of ECG and its transmission rate, regardless of admission control scheme. Thus, the admission control does not cause any additional computational cost.

Note that the achievable throughput in Eq (6) is derived without considering the collision under a reasonable assumption that an excessive ECG connection is not admitted by the admission control. However, the collision may happen with an insignificant probability even though the admission control maintains the number of ECG connections within the network capacity. For example, if two admitted nodes with the ECG application generate packets at the same time, and they determine the same initial backoff counter, the collision inevitably occurs among them. In order to minimize this possible collision, we devise a simple add-on scheme for the admission control. Let us denote *T*_*int*_ as the packet generation interval of ECG application and denote *t*_*i*_ as the time when the ECG connection *i* is admitted. We define Δ*t*_*i*_(> 0) as the timing offset of ECG connection *i* within *T*_*int*_:

$$\Delta t_{i}=\text{mod}\left(t_{i},T_{int}\right),$$

where mod(*x*, *y*) is a function that returns the modulus after dividing *x* by *y*. If the AP receives a new acceptable ECG connection request at the time of *t*_*k*_, it checks the difference between Δ*t*_*k*_ and Δ*t*_*i*_(∀*i* ≠ *k*). If this difference is lower than CW_MAX_ECG ⋅ *T*_slot_ for any *i*(≠ *k*), the AP does not immediately transmit the admission response message, but it delays the admission by the time of *d*(> 0) such that:

$$\begin{array}{cc} & \Delta t_{k}^{{}^{\prime }}=\text{mod}\left(t_{k}+d,T_{int}\right),\\ & {\text{min}}_{i\neq k}\left|\Delta t_{k}^{{}^{\prime }}-\Delta t_{i}\right|>\text{CW}\_\text{MAX}\_\text{ECG}\cdot T_{\text{slot}}. \end{array}$$ (7)

Then, the AP maintains the timing offset of ECG connection *k* as the updated value of $\Delta t_{k}^{\prime }$. If there is no *d* satisfying Eq (7), the AP accepts the connection request without the artificial delay. However, this probability is negligible, because the ECG connection request occurs at a random time, $N_{ECG}^{MAX}$ is a few tens, *T*_*int*_ is in the order of a hundred milliseconds, and CW_MAX_ECG ⋅ *T*_slot_ is much less than one millisecond. In this way, the delayed admission of ECG connection according to Eq (7) spreads the timing offset to minimize the collision among ECG connections.

## Performance evaluation

We implement the conventional IEEE 802.11e, absolute priority scheme [17], and the proposed adaptive AIFS algorithm with ns-2 simulator [21] and compare their performance in various aspects via simulations.

### Simulation configuration and performance indices

We consider three types of traffic as in Table 4 according to [4]. For the realistic simulation, we emulate the data of ECG traffic from *MIT-BIH arrhythmia* database [22], which is obtained at Boston’s Beth Israel Hospital for research purpose. We also consider that all the nodes can sense each other, i.e., there is no hidden node, and set the PHY and MAC parameters according to IEEE 802.11 standard; *T*_slot_ is set to 20 *μs*, *R*_*min*_ is set to 1 Mb/s, and the size of PHY header, MAC header, and ACK frame are set to 15 bytes, 20 bytes, and 14 bytes, respectively. The simulation results are averaged for 50 runs with different random seeds and the simulation time is set to 4,000 seconds.

**Table 4:** Traffic characteristics in the simulation study.

Traffic type	Alarm	ECG	Data
Access category	AC_VO (high)	AC_VI (middle)	AC_BE (low)
Traffic model	on/off (exponential)	periodic	greedy
Traffic parameters	average on time: 1 s	average rate: 25.6 Kb/s	230mmalways have data to transmit
	average event/hour: 3.6	inter-arrival time: 200 ms	
Packet size	640 Byte	640 Byte	1500 Byte
Transport protocol	UDP	UDP	TCP (New Reno)
Transmission rate	1 Mb/s		

**Fig 6:**
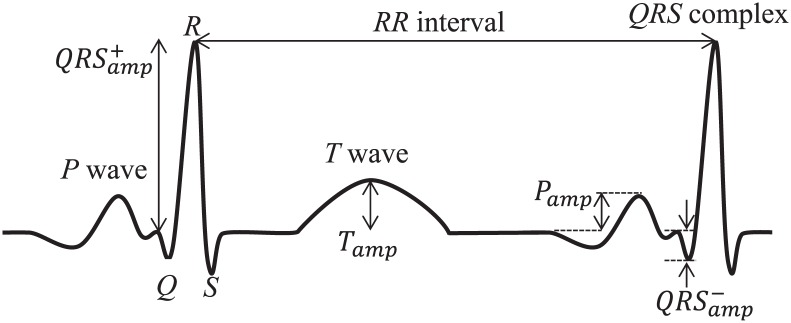
Features of ECG signal.

As performance indices for alarm traffic and ECG traffic, we define *ν*_*ALR*_ and *ν*_*ECG*_, which are the rates of valid alarm and ECG packets whose delivery delay do not exceed the maximum allowable delay of MAX_DLY_ALR and MAX_DLY_ECG, among the total number of alarm and ECG packets transmitted, respectively. In the meantime, we introduce a more advanced medical-grade performance index, weighted diagnostic distortion (WDD) [23] for real-time ECG monitoring application. The conventional QoS metrics, such as delay and loss, cannot reflect the importance of medical information contained in a packet. WDD measures and represents the level of data distortion quantitatively by comparing the original and received ECG signals, which can be characterized by the interval and amplitude of specific waves as shown in Fig 6. It has the ideal value of zero if there is no distortion at all, and its value increases up to one as the degree of distortion becomes more severe. Let us define *f* and $\hat{f}$ as a vector of key diagnostic parameters in the original and received ECG signals, respectively. Furthermore, we define Δ*f* as the normalized difference vector between *f* and $\hat{f}$:

$$\Delta f={\left[\Delta f_{1},\Delta f_{2},\ldots ,\Delta f_{m}\right]}^{T}\;\text{with}\;\Delta f_{i}=\frac{|f_{i}-{\hat{f}}_{i}|}{\text{max}\{|f_{i}|,|{\hat{f}}_{i}|\}},$$

where *f*_*i*_ and ${\hat{f}}_{i}$ are the *i*-th element in the vector of *f* and $\hat{f}$, respectively, and *m* is the size of the vector, i.e., the number of diagnostic parameters. The value of WDD is calculated based on Δ*f* as follows:

$$\text{WDD}\left(f,\hat{f}\right)=\Delta f^{T}\frac{\Lambda }{tr(\Lambda )}\Delta f,$$

where Δ*f*^*T*^ is the transpose of Δ*f* and Λ is a diagonal weighting matrix for diagnostic parameters:

$$\Lambda =\text{diag}\left[\lambda_{i}\right];\lambda_{i}>0\;\text{where}\;i=1,2,\ldots ,m,$$

and *tr*(Λ) is the trace of matrix Λ, i.e., $tr(\Lambda )=\sum_{i=1}^{m}\lambda_{i}$. We define $\bar{WDD}$ as the complementary WDD, i.e., $\bar{WDD}\;=\;1\;-\text{ WDD}$, in order to evaluate and compare the performance in the positive aspect, as similar to *ν*_*ALR*_ and *ν*_*ECG*_.

### Performance comparison

First, we evaluate and compare the medical-grade QoS of alarm and ECG traffic. Note that, in this subsection, the admission control is intentionally disabled in the proposed scheme in order to focus on the performance of the adaptive AIFS scheme; the admission control will be considered in the next subsection. We set the number of nodes with alarm and data traffic as 5 and 20, respectively, and change the number of nodes with ECG traffic from 1 to 25, by considering the capacity analysis of ECG applications in Section 4.2.1. Hereafter, we denote EDCA, ABS_PRIO, and ADP_AIFS as the conventional IEEE 802.11e, the absolute priority scheme [17], and the proposed scheme, respectively.

**Fig 7:**
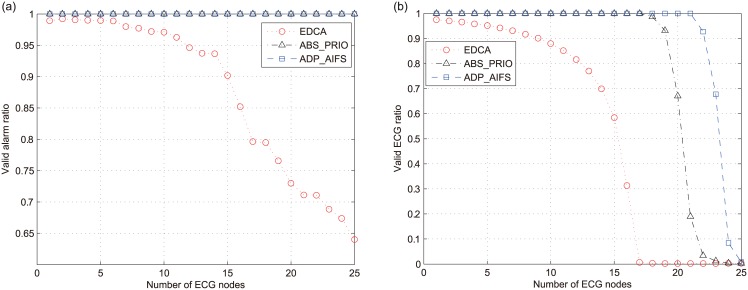
Ratio of valid packets over total packets received. (a)Alarm (*ν*_*ALR*_). (b)ECG (*ν*_*ECG*_).

Fig 7(a) and 7(b) show the ratio of valid packets for alarm and ECG traffic, i.e., *ν*_*ALR*_ and *ν*_*ECG*_, respectively, when the number of nodes with ECG traffic (*N*_*ECG*_) increases from 1 to 25. From Fig 7(a), we can observe that the QoS of highest-priority alarm traffic is liable to be degraded in EDCA owing to the increase of lower-priority ECG traffic. On the other hand, ABS_PRIO and ADP_AIFS completely avoid the delayed alarm packets, regardless of the load of ECG traffic; that is *ν*_*ALR*_ is one for the entire range of *N*_*ECG*_.

We can observe the superior performance of ADP_AIFS in terms of *ν*_*ECG*_ from Fig 7(b). In the case of EDCA, *ν*_*ECG*_ is not one even when *N*_*ECG*_ = 1, and it exponentially decreases to 0.3 when *N*_*ECG*_ = 16 and eventually reaches zero as long as *N*_*ECG*_ ≥ 17. Along with the result in Fig 7(a), this result reconfirms the serious problem of EDCA in assuring the medical-grade QoS. ABS_PRIO effectively mitigates this problem when the load of ECG traffic is not high. However, as *N*_*ECG*_ increases exceeding 19, *ν*_*ECG*_ begins to abruptly decrease. On the other hand, ADP_AIFS completely assures that *ν*_*ECG*_ = 1 as long as *N*_*ECG*_ < 22. The results in Fig 7 confirm that ADP_AIFS effectively assures the strict medical-grade QoS of alarm traffic while increasing the capacity of lower-priority ECG traffic.

**Fig 8:**
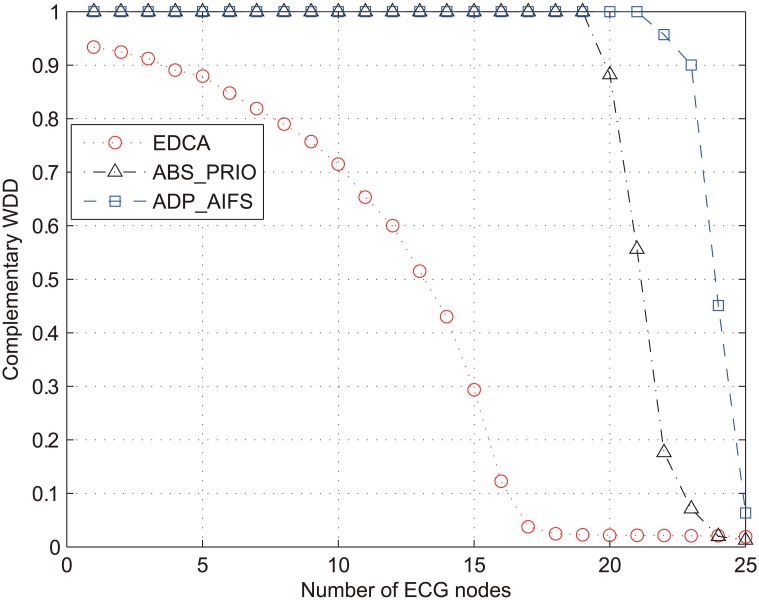
Complementary weighted diagnostic distortion of ECG packets ($\bar{WDD}$).

Fig 8 compares the performance of several schemes in terms of $\bar{WDD}$ of ECG traffic. In calculating WDD, we used the three diagnostic parameters of ECG signal *f*_1_ = RR_interval_, $f_{2}={\text{QRS}}_{\text{amp}}^{+}$, and $f_{3}={\text{QRS}}_{\text{amp}}^{-}$ in Fig 6, which are the most critical features in the medical diagnosis, and set the same weight for them, i.e., *λ*_1_ = *λ*_2_ = *λ*_3_ = 2, according to the guideline in [23]. Similar to the results in Fig 7, the results in Fig 8 confirms the outstanding performance of ADP_AIFS. The values of $\bar{WDD}$ in EDCA and ABS_PRIO drops below 0.9 when *N*_*ECG*_ ≥ 5 and 20, respectively. However, ADP_AIFS maintains $\bar{WDD}$ greater than 0.9 as long as *N*_*ECG*_ ≤ 23.

**Fig 9:**
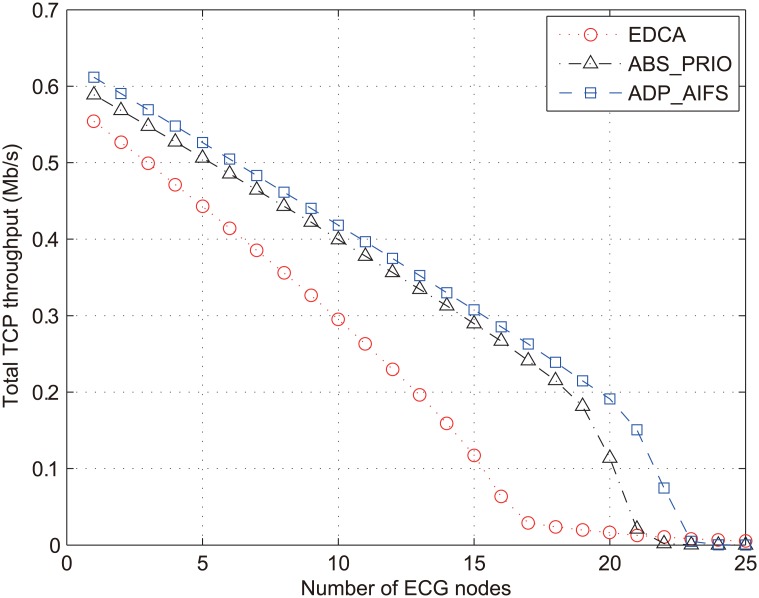
Total throughput of data traffic (*TH*_*DAT*_).

Now, we focus on the performance of the lowest-priority data traffic. Fig 9 shows the total throughput achieved by 20 nodes with data traffic, denoted as *TH*_*DAT*_ with respect to *N*_*ECG*_ ranging from 1 to 25. As was expected, *TH*_*DAT*_ decreases as *N*_*ECG*_ increases in all the schemes. This is because the channel access opportunity of data traffic decreases as the load of higher-priority traffic increases. In the aspect of channel access competition with higher-priority traffic, EDCA grants more access opportunities to data traffic thanks to the shorter AIFS, compared to ABS_PRIO, as indicated in Fig 4. Therefore, it can be expected that EDCA increases *TH*_*DAT*_, compared to ABS_PRIO. However, this is not the case, as shown in Fig 9, i.e., *TH*_*DAT*_ of EDCA is rather smaller than that of ABS_PRIO in the most range of *N*_*ECG*_. This result can be explained from two points. The frequent channel access of data traffic in EDCA may lead to frequent collisions, and the collision doubles the value of CW according to the BEB mechanism, which, in turn, decreases the channel access opportunity for data traffic. On the other hand, the delivery delay of data packet tends to be increased because of retransmission of collided data packet. Therefore, the round-trip-time of TCP connection increases and its throughput decreases according to the TCP congestion control mechanism. Conversely, less aggressive channel access of data traffic in ABS_PRIO contributes to the increase of *TH*_*DAT*_. ADP_AIFS adjusts the opportunity for channel access of data traffic depending on the QoS measurement of higher-priority traffic. Fig 9 shows that ADP_AIFS maintains the highest value of *TH*_*DAT*_, regardless of *N*_*ECG*_. Especially when the network is almost saturated, for example, *N*_*ECG*_ is between 17 and 21, ADP_AIFS increases *TH*_*DAT*_ by more than 11.9 and 7.2 times, compared to EDCA and ABS_PRIO, respectively.

Table 5 compares several performance indices (*ν*_*ALR*_, *ν*_*ECG*_, $\bar{WDD}$, and *TH*_*DAT*_) of EDCA, ABS_PRIO, and ADP_AIFS when *N*_*ECG*_ = 10 and *N*_*ECG*_ = 20. Note that the network is considered to be hardly saturated when *N*_*ECG*_ = 10, whereas it is considered to be almost saturated when *N*_*ECG*_ = 20. Moreover, Table 5 lists the *relative gain* of ADP_AIFS against EDCA and ABS_PRIO, which is defined as the difference between values of performance indices in ADP_AIFS and the other comparative scheme divided by the value of performance indices in the comparative scheme. When *N*_*ECG*_ = 10, ADP_AIFS slightly outperforms EDCA in terms of *ν*_*ALR*_, but greatly improves $\bar{WDD}$ and *TH*_*DAT*_ by more than 40%. The relative gain of ADP_AIFS over ABS_PRIO is close to zero, i.e., there is no notable difference between the performances of ADP_AIFS and ABS_PRIO, when the network is free from overload. However, when the network becomes saturated, i.e., *N*_*ECG*_ = 20, the relative gain of ADP_AIFS becomes outstanding. Compared to EDCA, ADP_AIFS improves $\bar{WDD}$ and *TH*_*DAT*_ by about 49 and 8.5 times. Similarly, ADP_AIFS improves *ν*_*ECG*_ and *TH*_*DAT*_ by about 14% and 73%, respectively, compared to ABS_PRIO. These results confirm that ADP_AIFS strictly assures the medical-grade QoS of high-priority traffic regardless of network load, and at the same time, it notably improves the performance of low-priority traffic especially as the network load increases.

**Table 5:** Performance comparison and relative gain of the proposed scheme over the existing schemes.

	*N*_*ECG*_ = 10				*N*_*ECG*_ = 20			
Performance indices	*ν*_*ALR*_	*ν*_*ECG*_	$\bar{WDD}$	*TH*_*DAT*_ (Mb/s)	*ν*_*ALR*_	*ν*_*ECG*_	$\bar{WDD}$	*TH*_*DAT*_ (Mb/s)
Absolute value								
EDCA	0.97	0.88	0.71	0.29	0.73	0	0.02	0.02
ABS_PRIO	1	1	1	0.40	1	0.67	0.88	0.11
ADP_AIFS	1	1	1	0.42	1	1	1	0.19
Relative gain								
over EDCA	3%	14%	41%	45%	37%	∞	4900%	850%
over ABS_PRIO	0%	0%	0%	5%	0%	49%	14%	73%

**Fig 10:**
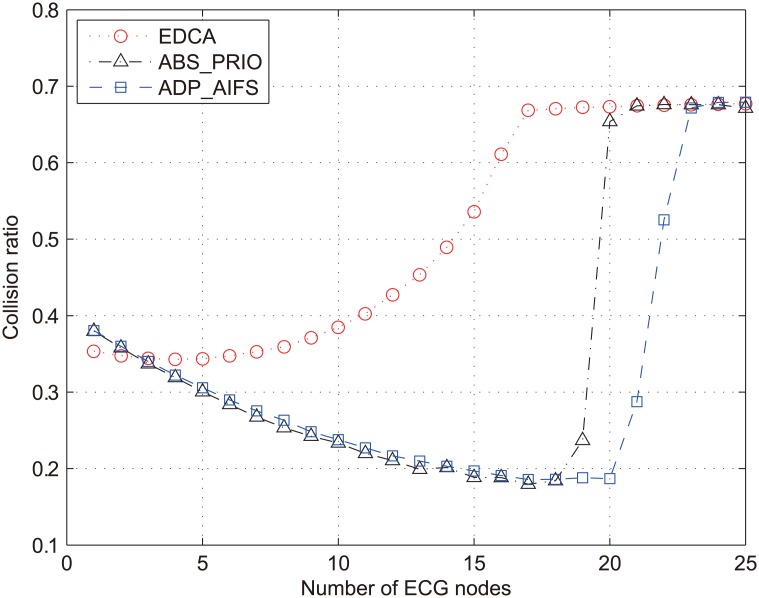
Collision ratio of total packets.

Fig 10 compares the collision ratio (*γ*_*coll*_) of the three schemes, which proves the improvement of *TH*_*DAT*_ in the proposed scheme. Note that collisions in all the type of traffic are accounted in measuring *γ*_*coll*_. As shown in Fig 10, *γ*_*coll*_ of IEEE 802.11e increases with respect to the increase of *N*_*ECG*_, which results in the decrease of *TH*_*DAT*_. By contrast, in ABS_PRIO and ADP_AIFS, *γ*_*coll*_ decreases as *N*_*ECG*_ increases up to 18 and 20, respectively. This is because both schemes serve low-priority data packets conservatively so that low-priority packets do not suffer from severe collisions, and the increase of *N*_*ECG*_ further restricts the channel access opportunity for low-priority packets. In the range of 17 ≤ *N*_*ECG*_ ≤ 21, ADP_AIFS remarkably decreases *γ*_*coll*_ by up to about 3.6 and 3.5 times, compared to EDCA and ABS_PRIO, respectively.

### Effect of admission control

In this subsection, we focus on the effect of admission control. Here, we only consider ECG traffic and change the number of ECG nodes with respect to time so that the network is overloaded during a specific time interval.

**Fig 11:**
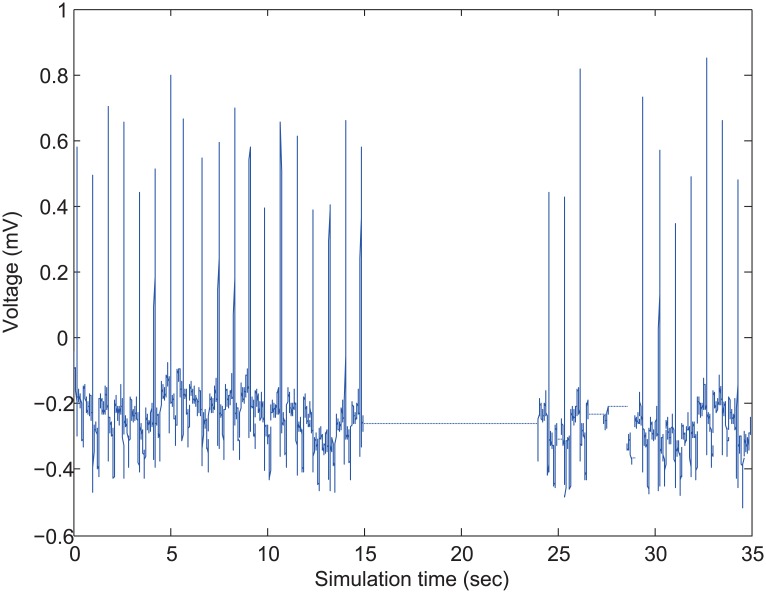
Received ECG traffic.

Firstly, we observe how the received ECG signal is affected by the network overload when the admission control is not applied. Fig 11 shows the actual received ECG signal from a specific ECG node. In this simulation, *N*_*ECG*_ is increased from 25 to 28 at *t* = 15 sec, and it is returned to 25 at *t* = 17 sec, so that the network is overloaded during *t* = [15–17] sec. From Fig 11, we can observe that even though the network is overloaded for only 2 seconds of *t* = [15–17] sec, the node does not receive any ECG packet until *t* = 24 sec. In the detailed analysis, the network overload distorts the ECG signal extremely so that it becomes useless at all. The result in Fig 11 strongly supports the necessity of admission control to deal with the network overload for maintaining the acceptable medical-grade QoS.

Next, we compare the delay of ECG packets in two cases with and without the admission control in our scheme. For this purpose, we consider 30 ECG nodes and divide them into three groups and let nodes in each group start to transmit packets at *t* = 5 sec, 10 sec, and 50 sec. The nodes in the first group terminates packet transmissions at *t* = 300 sec, while the nodes in the second and third groups continue to transmit packets until the end of simulation. In this configuration, the network is highly overloaded without the admission control during *t* = [50–300] sec. We set the parameters of admission control as $N_{ECG}^{MAX}\;=\;25$ and Δ*N*_*ECG*_ = 0, by considering the simulation configuration.

**Fig 12:**
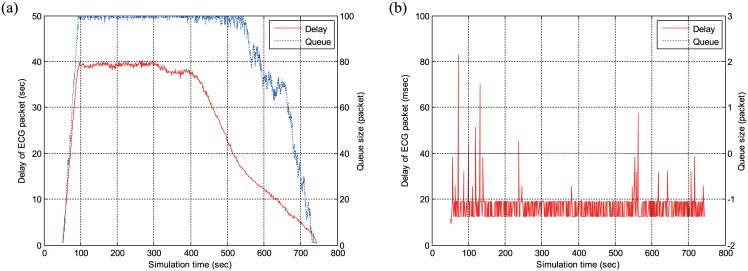
Delay of ECG packets and queue length with and without the admission control. (a)Without admission control. (b)With admission control.

Fig 12(a) plots the average delay of ECG packets transmitted by all the nodes, together with the queue length in a specific node, when the admission control is disabled. From Fig 12(a), we can observe that the delay starts to sharply increase at *t* = 50 sec when the network becomes overloaded, maintained at about 39 sec during *t* = [90–400] sec, and slowly decreases from *t* = 400 sec to *t* = 750 sec. We can infer the reason for the significant increase in the delay without the admission control by observing the queue length. When the network is saturated from *t* = 50 sec owing to lack of admission control, the queue starts to grow and its length reaches the maximum value of 100 packets. This implies that many ECG packets are dropped because of buffer-overflow. Even after the network is not overloaded at *t* = 300 sec, the queue length does not start to decrease until *t* = 550 sec. In other words, it takes a few hundred seconds to resolve the network congestion resulting from overload.

Fig 12(b) confirms that this problem can be effectively dealt with by the admission control. It is important to note that the scale of delay in Fig 12(b) is milliseconds, while that in Fig 12(a) is seconds. When the admission control is applied, even the maximum delay is about 80 msec, which is much smaller than the MAX_DLY_ECG (= 200 ms), i.e., there is no QoS violation for ECG packets at all. Moreover, the average delay is about 16.5 msec, which is smaller than the case without admission control by three orders of magnitude. On the other hand, the queue length, measured as the number of backlogged packets in the queue at the time of a packet arrival, is definitely zero for the whole simulation time. Therefore, as soon as the ECG packet arrives, it can be served immediately without any queueing delay. From the results in Fig 12, we can conclude that the admission control is absolutely required to avoid the severe degradation of medical-grade QoS due to network overload and buffer-overflow and that the significant increase of delay without the admission control primarily results from the queuing delay.

## Conclusion

In this study, we have shown that the conventional IEEE 802.11e protocol is insufficient to guarantee the required QoS of medical applications in wireless healthcare systems because of priority inversion and collision. To cope with this problem, we have proposed two schemes; adaptive AIFS scheme and admission control scheme. The former tunes the AIFS of lower-priority traffic based on the QoS measurement of high-priority traffic. Thus, it provides the differentiated near-absolute priority for high-priority traffic, at the same time, it does not unnecessarily decrease the channel access opportunity for low-priority traffic. The latter has been proposed to avoid the performance degradation owing to network overload and frequent collisions. Our simulation results have shown that the proposed mechanism strictly assures the medical-grade QoS for high-priority medical traffic while significantly improving the throughput of low-priority non-medical traffic.

## References

[1] GE Healthcare. Product features-wireless technologies. Available from: http://www.gehealthcare.com.

[2] Philips. Patient monitoring–intellivue telemetry system. Available from: http://www.medical.philips.com.

[3] Welch Allyn. Micropaq wearable monitor. Available from: http://www.welchallyn.com.

[4] Baker SD, Hoglund DH. Medical-grade, Mission-critical Wireless Networks. IEEE Engineering in Medicine and Biology Magazine. 2008;27(2):86–95. 10.1109/EMB.2008.915498 18463024

[5] Zhang Y, Sun L, Song H, Cao X. Ubiquitous WSN for Healthcare: Recent Advances and Future Prospects. IEEE Internet of Things Journal. 2014;1(4):311–318. 10.1109/JIOT.2014.2329462

[6] Du Q, Zhao W, Li W, Zhang X, Sun B, Song H, et al Massive Access Control Aided by Knowledge-extraction for Co-existing Periodic and Random Services over Wireless Clinical Networks. Journal of Medical Systems. 2016;40(7):1–8. 10.1007/s10916-016-0506-5 27240842

[7] Jiang Y, Song H, Wang R, Gu M, Sun J, Sha L. Data-Centered Runtime Verification of Wireless Medical Cyber-Physical System. IEEE Transactions on Industrial Informatics. 2016;preprint. 10.1109/TII.2016.2573762

[8] Zhang Y, Liu S, Zhang R, Wei W, Song H, Li W, et al. A New Multi-service Token Bucket-shaping Scheme Based on 802.11e. In: 2015 International Conference on Identification, Information, and Knowledge in the Internet of Things (IIKI). IEEE; 2015: 197–200.

[9] Luo H, Shyu ML. Quality of Service Provision in Mobile Multimedia-a Survey. Human-centric Computing and Information Sciences. 2011;1(1):1–15. 10.1186/2192-1962-1-5

[10] Romdhani L, Ni Q, Turletti T. Adaptive EDCF: Enhanced Service Differentiation for IEEE 802.11 Wireless Ad-Hoc Networks In: 2003 Wireless Communications and Networking (WCNC). IEEE; 2003;2:1373–1378.

[11] Deb B, Hartman MJ. Distributed Optimization of Contention Windows in 802.11e MAC to Provide QoS Differentiation and Maximize Channel Utilization. In: 2012 IEEE International Symposium on a World of Wireless, Mobile and Multimedia Networks (WoWMoM). IEEE; 2012: 1–7.

[12] Pudasaini S, Shin S. QoS Provisioning in CSMA/iCA Based Medium Access Control Protocol for WLAN. In: 2012 Fourth International Conference on Ubiquitous and Future Networks (ICUFN). IEEE; 2012: 340–345.

[13] Freitag J, da Fonseca NL, de Rezende JF. Tuning of 802.11e Network Parameters. IEEE Communications Letters. 2006;10(8):611–613. 10.1109/LCOMM.2006.1665127

[14] Arora A, Yoon SG, Choi YJ, Bahk S. Adaptive TXOP Allocation Based on Channel Conditions and Traffic Requirements in IEEE 802.11e networks. IEEE Transactions on Vehicular Technology. 2010;59(3):1087–1099. 10.1109/TVT.2009.2031677

[15] Andreadis A, Zambon R. Improving QoS Performance in IEEE 802.11e Under Heavy Traffic Loads. International Journal of Wireless Information Networks. 2012;19(1):49–61. 10.1007/s10776-011-0162-0

[16] Ghazvini M, Movahedinia N, Jamshidi K. GTXOP: A Game Theoretic Approach for QoS Provisioning Using Transmission Opportunity Tuning. PLoS ONE. 2013;8(5):e62925 10.1371/journal.pone.0062925 23650539 PMC3641108

[17] Lee H, Park KJ, Ko YB, Choi CH. Wireless LAN with Medical-grade QoS for E-healthcare. Journal of Communications and Networks. 2011;13(2):149–159. 10.1109/JCN.2011.6157414

[18] Shojafar M, Cordeschi N, Baccarelli E. Energy-efficient Adaptive Resource Management for Real-time Vehicular Cloud Services. IEEE Transactions on Cloud Computing. 2016: 1 10.1109/TCC.2016.2551747

[19] Baccarelli E, Cordeschi N, Mei A, Panella M, Shojafar M, Stefa J. Energy-efficient Dynamic Traffic Offloading and Reconfiguration of Networked Data Centers for Big Data Stream Mobile Computing: Review, Challenges, and a Case Study. IEEE Network. 2016;30(2):54–61. 10.1109/MNET.2016.7437025

[20] Laufer R, Kleinrock L. On the Capacity of Wireless CSMA/CA Multihop Networks. In: 2013 Proceeding of INFOCOM. IEEE; 2013: 1312–1320.

[21] The network simulator (ns-2). Available from: http://www.isi.edu/nsnam/ns/.

[22] Moody GB, Mark RG. The Impact of the MIT-BIH Arrhythmia Database. IEEE Engineering in Medicine and Biology Magazine. 2001;20(3):45–50. 10.1109/51.932724 11446209

[23] Zigel Y, Cohen A, Katz A. The Weighted Diagnostic Distortion (WDD) Measure for ECG Signal Compression. IEEE Transactions on Biomedical Engineering. 2000;47(11):1422–1430. 10.1109/TBME.2000.880093 11077735

